# Pediatric Market Access: A Qualitative Study

**DOI:** 10.1007/s43441-023-00601-6

**Published:** 2024-01-03

**Authors:** Lieke Maas, Angelika Joos, Mickael Hiligsmann

**Affiliations:** 1 Department of Health Services Research, Care and Public Health Research Institute (CAPHRI), Maastricht University, P.O Box 616, 6200 MD Maastricht, The Netherlands; 2 Global Regulatory Policy, Merck Research Laboratories, MSD (Europe) Inc., Brussels, Belgium

**Keywords:** Pediatrics, Pediatric medicines, Market access, Reimbursement, Pediatric regulation

## Abstract

**Objectives:**

This qualitative study aims to analyze current PM regulation and market access requirements and proposes potential solutions to mitigate current challenges.

**Methods:**

Twenty-two semi-structured interviews were conducted with experts from pharmaceutical industry, regulatory authorities, national health technology assessment (HTA) bodies, pediatricians, and academia from the Netherlands (NL), Germany (DE), the United Kingdom (UK), and France (FR) to get insight into the pediatric research, the regulatory and reimbursement processes, challenges, and solutions. Themes for further testing were developed on how to facilitate pediatric market access. Atlas.ti 9 was used to analyze the findings.

**Results:**

Heterogeneity in requirements for the European Medicines Agency (EMA) and HTA approvals are noted. By example, DE grants direct reimbursement after regulatory approval, the other countries require additional reimbursement which generate delays and challenges in patient access after marketing authorization. Key components in facilitating PM market access include multi-stakeholder collaboration, transparency, patient representatives, informed consent guidance, real-world evidence, and appropriate clinical trial designs. Pricing models based on the economic capabilities of individual countries could further reduce delays and challenges in market access. The additional specific pediatric incentives should be taken as best practice to encourage innovation in pediatric conditions.

**Conclusion:**

This study highlights differences in requirements for regulatory and reimbursement approval, along with international differences in pricing and reimbursement procedures for pediatric market access.

## Introduction

There has been increasing attention to market access of pediatric medicines (PM). PM are generally developed alongside adult drug development. However, most of them do not get immediate approval and when children become ill, doctors often prescribe ‘off-label’ medicines, i.e., medicines that have not been approved for use in children [1]. Two cohort studies demonstrated that over half of all pediatric patients receive off-label/unlicensed drug prescriptions during their hospital stay [2]. Turner et al. demonstrated that about 1/3 to 1/2 of pediatric adverse drug reactions occur during off-label prescriptions, and pediatric patients treated with off-label prescriptions have a six-fold higher risk of adverse drug reaction compared to on-label prescriptions (7.3 vs 1.2 percent) [3]. These findings are supporting the medical and scientific paradigm shift toward treating pediatric patients with approved pediatric drugs while shifting away from using effectiveness and safety assumptions based on adult data [4].

Only eight to fifteen percent of PM development programs survive from pre-clinical development to the pharmacy shelf [5]. Challenges include small patient populations, ethical considerations for clinical trials, age-appropriate formulations, and lack of available pediatric options [5]. To overcome these challenges, the European Union (EU) adopted a regulation in 2007 with three objectives: more medicines for children, better product information, and more pediatric research [6]. The regulation obliges companies to scrutinize every new drug under development for a child-friendly version, to increase data on children, which is done through a Pediatric Investigation Plan (PIP) [6]. A PIP outlines a development program on how to obtain appropriate data in children including a child-friendly formulation that is appropriate for regulatory approval. This PIP needs to be presented by the developer and approved by regulators before the completion of the human pharmacokinetics studies in adults of the concerning drug [6]. Alongside this PM development obligation, the regulation offers several benefits for PM, i.e., free regulatory scientific advice and protocol assistance from the European Medicines Agency (EMA). A supplementary protection certificate (SPC) offers up to five years extension of the patent protection to compensate developers for long development and regulatory approval times. If a PIP has been completed, this period can be extended by 6 months. Orphan medicines that are developed for children receive an additional two years of market exclusivity in addition to the ten-year market exclusivity of adult orphan drugs. Lastly, the PUMA, which provides ten-year market protection period for a pediatric indication for a product that is no longer protected by an SPC [7]. These rewards are not cumulative and there are no incentives in the regulation that facilitate national reimbursement or guarantee patient access. The lack of regulations and incentives for market access to PM harm the health of children primarily promoting use of off-label/unlicensed products and delaying access to appropriate medicines and formulations.

While pediatric regulations successfully created more ‘push’ incentives for researchers to invest in PM development, challenges regarding efficiency and market access to PM have remained. These include the lack of research meeting pediatric needs, inefficient timely completion of PIPs due to unavailability of suitable patients for clinical trials, and insufficient collaboration and transparency of PM research [6, 8]. Therefore, there is a need for novel mechanisms to ensure timely completion of pediatric studies included in the PIP [4].

Although literature already identifies challenges regarding PM regulation and market access, to date there is no overview available and only limited knowledge on initiatives needed to mitigate these challenges. This study aims to analyze the PM regulation and market access requirements in selected countries and proposes solutions to mitigate the current challenges. This explorative study consists of a qualitative approach aiming to gain further insights into challenges of PM access, especially differences in reimbursement pathways. In particular, this study analyses how PM development can be stimulated through reimbursement incentives within the Netherlands (NL), Germany (DE), the United Kingdom (UK), and France (FR). This research is timely especially with recent development of regenerative medicines (such as cell therapies, gene therapies, gene editing constructs) that are primarily targeted for pediatric populations.

## Materials and Methods

### Study Design

The study design entails a qualitative study with a constructivism/phenomenological approach. Phenomenological research can be explained as comparing findings from the qualitative study to quantitative findings in literature to determine current knowledge of a phenomenon, in this case market access of PM. This study design was chosen to scrutinize the PM regulation effectiveness and market access requirements and enables comparisons of findings from qualitative and quantitative results to determine current knowledge of PM market access.

### Data Collection

Data were collected through semi-structured interviews conducted between April and June 2022. This form of interviewing was chosen as it allows elaboration on answers [9]. Interviews were executed individually via MS Teams. The interview, transcription, and coding were performed in English unless interviewees requested it in Dutch. Audiotaping was used to enable interview transcriptions.

The target population included experts with either academic/clinical, regulatory, or reimbursement experience in the field of PM. Both regulatory and reimbursement interviewees included public agency perspectives from EMA or Health Technology Assessment (HTA) bodies and private industry perspectives from experts working in regulatory affairs and market access departments. We targeted to interview one industry reimbursement and one HTA representative for each country, two clinical and/or scientific/academic representatives, and two regulatory representatives. It was important that the interviewees had at least five years of experience in their field. Four countries of focus were selected based on their differences and leading roles in health care, namely NL, DE, the UK, and FR. In total, ten external (different companies) and twelve internal (pharmaceutical company) experts participated. More specifically, two (ex) pediatricians, three (two ex, one in) researchers, four regulatory representatives (two in, two ex), and thirteen reimbursement representatives participated. The reimbursement representatives included four HTA experts (all ex: one EU, two NL, one UK), and nine industry market access experts (one ex, eight in: two EU, two NL (one ex), one DE, one UK, three FR).

Experts were interviewed for twenty-thirty minutes. Prior to the interviews, themes with questions were identified and reviewed for relevance by three experts in the field. Initial questions were formulated with help of a regulatory expert (Table 1). An academic supervisor was also involved in the process. A scoping review was conducted to evaluate the current state of literature during preparation of the questionnaire and alongside the study’s findings. Themes include challenges of PM regulatory processes, PM reimbursement incentives, benefits and challenges of pediatric drug development, and themes for further testing. Participants were interviewed in their professional function and do not represent the view of their institutions with the aim to obtain their individual perspective on research, regulatory, and reimbursement processes regarding PM market access. Participants were informed about the study via an information letter, voluntary participation, confidentiality of their responses, and data recording via audiotaping. All participants consented to the study before the interview. The study complies with the General Data Protection Regulations of Maastricht University and was approved by the ethics committee of Maastricht University, REC-form FHML/HPIM/2022.049.

**Table 1.** Interview Questions Per Perspective.

Perspective	Questions
Clinical/academia (C)	(1) What are the challenges that you encounter as a pediatrician when it comes to providing the right drugs for your patients? (2) I have read that there is a lot of off-label use. Can you tell me a bit about the problems surrounding off-label use and the risks you are forced to take? (3) How do you determine the right treatment for a child? (4) Are there any other challenges or worries that I should be aware of when it comes to drug access for children? (5) What would be your recommendations or is there anything that you would like to see differently (and how) I the future to solve the current problems?
Regulatory/reimbursement (A)/(R)	(1) Are there any regulations in place on a national/EU level with regards to pediatric medicines? (2) Are there any incentives in the pediatric reimbursement procedure for pediatric medicines? What is required, on-label, formulary, HTA assessment? (3) What are the current hurdles or challenges to market access for novel pediatric medicines from a regulatory/research perspective? (Such as off-label use) (4) Which elements of the current procedure work well in practice? (5) What kind of additional (economic) incentives need to be instated in your opinion to solve these challenges? What are your recommendations to facilitate/enhance market access for pediatric medicines?

*HTA* Health Technology Assessment, *EU* European Union, *C* clinical/academic perspective, *A* regulatory perspective including agency, such as EMA and industry regulatory affairs, *R* reimbursement perspective including agency HTA bodies and industry market access.

### Data Analysis

Interviews were transcribed according to the smooth verbatim approach. A member check was acquired from the interviewee to ensure the quality of the thematic data analysis and interpretation. Although clinicians and researchers had some differences in their interview questions, as there was overlap in results it was decided to code according to stage of PM market access the result referred to (clinical/research, regulatory, or reimbursement stage) instead of per question or coding theme. Data analysis was executed by one researcher according to the deductive approach using Atlas.ti 9 coding tool. A constant comparative technique was used to compare the interviews and determine if concepts should be expanded or merged.

## Results

Twenty-two experts with clinical/academic, regulatory, and reimbursement expertise were interviewed. The main results (see Table 2) are classified according to benefits and challenges these experts have encountered in either the clinical/academic, regulatory, or reimbursement phase, including the reimbursement systems of NL, DE, UK, and FR, and experts’ themes for further testing.

**Table 2.** Summary of the Main Interview Findings Including the Number of Interviewees that Described These Opinions.

Market Access Stage			Summary of Specific Issue		C Stakeholders	A + R Stakeholders
Clinical and scientific stage	European level	Research	Ethical considerations in pediatric research		A, R	3 C
			Lack of research in the actual pediatric unmet needs		4 A, R	–
			Off-label use discourages clinical trial enrollment		3 A, R	–
			Longer trials are preferred for viability, however, increases burden on clinicians		A	–
			Age groups differ from one another, so generalization of data is not feasible		R	C
			Initiating trials hampered due to unfavorable safety profile			C
			Need that patients in clinical trials are receiving active treatment at some point		R	–
			Finding specific pediatric researchers and hospital units who are willing to do such trials		R	–
		Clinical	Consent provided by parents. Pediatricians need to balance the best option for the patient and for parents simultaneously			C
			Trials are too burdensome for clinicians, leading to preference of off-label prescription		–	2 C
			Errors occur when creating in situ formulations from adult indications		–	C
			Dose is prescribed based on weight; however, pediatric metabolism could also affect efficacy of drug		A	C
			Awful tasting drugs leads to patients refusing to take it		–	2 C
			Off-label use: lack of available licensed PMs leading to using comparators with possible different complications		–	2 C
			Self-administration by parents, risk to not overdose or underdose child		–	C
			Full responsibility of pediatrician for off-label prescription		A	2 C
Regulatory stage	European level	+	Pediatric regulations have delivered what was intended to deliver		3 A, R	–
			Pediatric Regulations are well established, discussion and understanding present between industry and agency ensuring global harmonization		A	–
			SPC works well, incentivizes well		3 A, R	–
			Joint clinical assessment of efficacy by EUnetHTA works well		–	C
		–	Regulatory is a long and complex process		A, R	2 C
			PUMA barely used, no link between PUMA and pricing and reimbursement		A	C
			No incentives to develop for children’s diseases not present in adults		A	2 C
			Counterintuitive approach: when indication of medicines is extended to children, price for medicine is decreased		A	–
			PIP does not guarantee approval of pediatric indication, high risk of lost money		R	C
			No multi-stakeholder dialogue, HTA bodies not involved		A, R	2 C
Reimbursement stage	European level		Adult drug prescription cheaper in some countries resulting in disregarding pediatric indication		R	–
			Difficult to prove cost-effectivity or added benefit for pediatric indication		R	–
			Not having the right comparator		R	–
			Heterogeneity in requirements for EMA and HTA approvals		4 R	–
	National level	FR	+	Pediatric indication price at least equal to adult indication price guaranteed	–	–
				Pediatric dossiers are prioritized nationally	–	–
				No particular reimbursement challenges	3 R	–
			–	Prioritization of pediatric dossier depends on availability of clinical experts for evaluation	R	–
				Low adult indication price could negatively influence pediatric indication	2 R	–
				Public awareness of specific national pediatric rules is lacking	2 R	–
		DE	+	No particular reimbursement challenges	R	–
				Direct reimbursement approval of pediatric indication after adult indication	R	–
			–	Proving additional benefit with additional data for pediatric indications are hard leading to lower price, especially if comparator is generic	R	–
				Value of pediatric formulations not taken into account enough	R	–
				No distinguishment between adult and pediatric indications	R	–
		NL	+	No particular reimbursement challenges	3 R	–
				Ethical and societal assessment of drug well appreciated; assessment assisted by scientific board	R	–
				Strong alliance with health institute and Ministry of Health to negotiate prices	R	–
				Kinderformularium works well in clinical practice	–	C
				In case of adult indication present, acceleration of reimbursement procedure for pediatric indication	R	–
			–	No distinguishment between adult and pediatric indications	R	–
				Insufficient acknowledgment of present pediatric need	R	–
				Lengthy reimbursement procedure	4 R	–
				Methodological challenges of measuring cost-effectiveness and QoL	2 A, R	–
		UK	+	No particular reimbursement challenges	1 in 1 ex R	–
				NICE incorporates public opinion	2 R	–
				Direct reimbursement approval of pediatric indication after adult indication	2 R	–
			–	NICE can quite restrictive	R	–
				Due to automatic pediatric approval, no independent pediatric research on pediatric indication is performed	2 R	–
				Methodological challenges of measuring cost-effectiveness and QoL	2 R	–
				Difficulty in assessing effectivity due to varying age groups and different comparators available in each age groups	2 R	–
				No distinguishment between adult and pediatric indications	2 R	–
Expert themes for further testing		Incorporate multi-stakeholder collaboration to understand perspectives of all parties involved			10 A, R	3 C
		Joint databases for post-approval and real-world evidence sharing			4 R	4 C
		Early dialogue with HTA bodies, preferably during PIP development			9 A, R	–
		Streamline communication between EMA and HTA bodies			5 A, R	3 C
		More recognition of industry efforts in addressing pediatric needs without an adult indication			4 R	–

The results from the questions in Table 1 were sorted according to which stage of the market access process the result referred to. The stakeholders’ responses have been divided by the two groups from table two C (= clinical/academic) stakeholders and A + R (= reimbursement and regulatory) stakeholders.

+ benefits, − challenges, *EUnetHTA* European Network for Health Technology Assessment, *PM* pediatric medicines *PUMA* Pediatric-Use Marketing Authorisations, *QoL* quality of life, *NICE* National Institute for Health and Care Excellence, *HTA* Health Technology Assessment, *SPC* Supplementary Protection certificate, *EMA* European Medicines Agency, *C* clinical/academic perspective, *A* Regulatory perspective including agency, such as EMA and industry regulatory affairs, *R* reimbursement perspective, including agency HTA bodies and industry market access.

### Benefits and Challenges in Pediatric Drug Development

#### Clinical and Scientific Phase

It is widely known that effective treatments are still lacking to address many pediatric unmet medical needs. Almost all regulatory experts acknowledge that in addition to the regulations to protect patients sufficiently, several challenges remain in conducting clinical research with pediatric patients. The first challenge entails ethical considerations like the burden on a patient population who cannot provide consent themselves, rely on parental permissions, or are only able to provide children’s assent. This results in additional burden on parents and biases introduced in research. One clinical/academic expert expressed extra considerations pediatricians experience besides considering the needs of the child, such as examining how rational the parents are in providing consent. Secondly, several experts, from both clinical/academic and regulatory expertise, mentioned the challenge in finding researchers in specialized pediatric fields and large competition for recruitment of suitable participants into clinical trials within the small population group. As pediatric age groups differ immensely from adults and one another concerning drug administration and reaction, clinical trials are particularly challenging. Moreover, PM development seems often to focus on the corresponding adult indications and therefore missing indications solely present in children. Clinical trials are also not stimulated by off-label use due to the cost and burden this creates for clinical centers.

All pediatricians voiced the pressure they encounter when prescribing treatments off-label and the risk of using unproven medicines. Moreover, pediatricians expressed the challenge in finding similar available medicines when their preferred off-label drug is not available, which was also confirmed by reimbursement and regulatory experts. Unavailability of appropriate formulations affects the ability to safely prescribe medicine and for parents (who are responsible for administering the drug to their child correctly) to avoid under- or overdosing. Pediatric formulations are often created in situ from adult medication, while difference in pharmacokinetic processes of absorption, distribution, metabolism, and excretion may appear and are not always fully taken into account [10]. The willingness of a child to take the drug also depends on taste and its ease of administration, which is often not accounted for in off-label medicines.

#### Regulatory Phase (EMA Agency and Industry Regulatory Affairs)

All three regulatory experts recognize benefits of the specific regulatory framework for PM. All twenty-two experts agree that it is a very lengthy process. Regulatory and reimbursement experts are satisfied with the pediatric regulation as it encourages companies to develop pediatric indications alongside their adult programs. More specifically, one regulatory expert stated that because the regulations are well established in multiple jurisdictions, all stakeholders are well informed, which enhances global harmonization and stimulates discussion. A clinical/academic expert confirmed this view and further highlighted the potential for a joint clinical assessment through the new EU HTA Regulation. The SPC reward is also explicitly appreciated among experts, specifically in industry as it can compensate for additional mandatory efforts to develop pediatric indications. Although the PIP increases medicine development for children, one research and one regulatory expert highlighted that it is a lengthy process with no guarantee for compensation of mandatory investments.

There are two broad categories of challenges surrounding the pediatric approval procedure. Firstly, all regulatory experts acknowledged that the incentives for pediatric drug development are insufficient. These include the lack of incentives for pediatric indication development independent from adult indications, insufficient linkage between the PUMA incentive and pricing and reimbursement leading to continued off-label usage of cheaper products, and potential mandatory product price decrease triggered by a pediatric indication when industry broadens the scope of the adult indication. Secondly, experts from all perspectives stress the lack of well-organized multi-stakeholder dialogue to overcome the challenges. The experts explained that this could facilitate convergence of regulatory and reimbursement procedures, create cooperation, and raise awareness and understanding of different stakeholders’ objectives.

### Pediatric Regulations and Incentives

Several experts emphasized the importance of adequate additional national incentives for industry and more involvement of patients during strategic development discussions. When comparing the four different countries, several distinctions can be made.

#### The Netherlands

Although NL has an accelerated procedure for reimbursement of indications where adult indications are already approved, this is not specific to pediatric indications. Dutch healthcare institute “*Zorg Instituut Nederland”* (ZIN) experts, market access experts, and pediatricians state that the reimbursement system is complex and not specifically tailored to pediatrics. Pediatricians often prescribe medicines off-label using the “*kinderformularium,*” which provides aid when calculating the dosages for their patients based on adult data and indications. Pediatricians believe that off-label reimbursement is well organized within the country. However, once indications are studied and approved by regulatory agencies, several challenges arise. While the national HTA body believe that there are no challenges in the reimbursement system, industry and the *kinderformularium* do not confirm these statements. All four national experts, from the reimbursement and regulatory perspective, criticize the lengthy procedure for assessing complex dossiers. The interviews with the Dutch HTA experts revealed that this is due to methodological challenges, including uncertainty around long-term safety, challenges in the measurement of quality of life in children, and lack of comparators for pediatric indications. It is important to note that NL is a small country with a small pediatric population, making it less attractive for developers to launch medicines in NL. Despite these challenges, experts state their appreciation for good collaboration between the Dutch health institute (ZIN) and the Ministry of Health regarding the price negotiations, elaborate ethical and societal assessment performed by scientific boards, and ongoing efforts to streamline the national procedure based on EU approvals.

#### Germany

DE has a more straightforward reimbursement procedure. Approved pediatric indications are automatically reimbursed. DE does not reimburse off-label use, unlike the other countries. Every drug receives free pricing during the first year on the market to facilitate market access. The German reimbursement expert stated that there are no specific challenges for pediatric market access. However, there did remain a potential challenge when determining the benefit assessments for pediatric indications. As the pediatric population is small, data may be insufficient to support price negotiation after the free pricing period. The licensed indication is often compared to generic medicines with a very low price and feasibility to provide sufficient evidence in demonstrating additional clinical benefit to secure a higher price is low. A German HTA representative was not interviewed.

#### The United Kingdom

The UK does not distinguish between medicines for adults and children similarly to NL and DE. The reimbursement process involves patient representatives in the decisions, which was highlighted by all British experts [11]. Interviews revealed that NICE tends to factor in the challenges in obtaining data for pediatric conditions when making recommendations. All experts, from reimbursement and regulatory perspectives, confirmed that there is an automatic access for paediatric indications where the adult indication already has reimbursement. However, experts in HTA bodies in Europe and the UK expressed possible challenges in pediatrics regarding unavailability of treatment in different age groups. More specifically, because of different comparators in the treatment pathway at different ages, products may only be granted reimbursement in pediatric setting under the age of eighteen, so for patients their treatment access can change with age [12].

#### France

FR does not automatically approve pediatric indications subsequently to the EU approval, even when an adult indication has already been approved. However, unlike other countries, FR has a specific pediatric reimbursement procedure. All three French reimbursement experts experience no challenges in the national pediatric reimbursement system and highlight several incentives specifically available for paediatric indications: FR prioritizes evaluation of paediatric indications, allows pre-submission meetings for reimbursement prior to EU approval, and accelerates pricing negotiations from 180 to 75 days. FR provides an additional year of price stability for paediatric indications if an adult indication is already present. FR guarantees the same minimum price as for the adult indication and includes patient representatives in their reimbursement process [13, 14]. One reimbursement expert highlighted that the prioritization of the paediatric dossier evaluation is highly dependent on the availability of the pediatric expert assessors and awareness in industry of these incentives. A French HTA representative was not interviewed.

### Experts’ Themes for Further Testing

More than half of the interviewees, from all perspectives, recommended putting more emphasis on multi-stakeholder collaboration, such as including patient representatives. Industry should work together, and perspectives from all stakeholders must be clearly highlighted. Eight clinical/academic and regulatory experts highlighted the importance of collaborating in joint databases to enable more post-approval monitoring and the use of real-world evidence. Nine experts, from reimbursement and regulatory perspectives, highlighted the importance of early dialogue discussing paediatric development strategy within the PIP, including HTA bodies and possibly extending the mandatory scope of the PIP to the mechanism of action of a new molecule. At least two experts from each perspective emphasized critical need to streamline communication between EMA and HTA bodies. Four regulatory experts suggested better recognition of industry’s efforts in addressing pediatric needs without adult indications.

## Discussion

This study reveals that PM development efforts are often built on development programs of adult indications. Indications solely present in children are insufficiently developed. Although regulations are well established leading to global harmonization, product price decrease for adult indications triggered by new pediatric indications can be a disincentive. No distinction is made between reimbursement procedures for pediatric indications in all countries except FR. All countries except NL have limited off-label use resulting from either automatic reimbursement approval or due to other specific pediatric incentives that prevent off-label use. Discrepancy and heterogeneity in requirements for regulatory and HTA approval generate delays and challenges in developing further evidence after marketing authorization. Although all industry experts of the four different countries felt that their national HTA bodies were more understanding when assessing pediatric data compared to adult data, only FR has regulations in place that particularly incentivise PM.

Interviewees highlighted several ethical and practical challenges in pediatric research. Namely, providing study consent when participating in a clinical trial or varying effectivity of drugs in different age groups. These ethical concerns were confirmed in recent publications in the field of pediatric research [15]. Experts voiced concern regarding the lack of medicine development targeting the unmet needs of pediatric patients independently from adult indications. They highlighted the difficulty in performing clinical trials due to wide-spread use of off-label medicines. The off-label prescription of an adult medicine is often a cheaper, less burdensome option and serve as comparators for PM [16]. This continued off-label use discourages patients and industry to invest in clinical trials to appropriately label these indications for pediatric use.

EU pediatric regulation has reached its goal to facilitate PM development and availability for children. Specifically, the SPC reward was well appreciated among all experts. A study comparing data before and after implementation of the regulation confirms that the regulation has encouraged development of pediatric indications alongside the adult development [17]. Regulatory challenges identified by experts include long approval processes, insufficient incentives for pediatric indications that address pediatric needs independent from adult development, and lack of multi-stakeholder dialogues to align patient, scientific, regulatory, and reimbursement expectations. Literature confirms that a PIP does not guarantee a successful pediatric indication and that regulatory and reimbursement requirements differ, streamlined procedures are desired, contradictory or lacking incentives are present, and cooperation and acknowledgment of the importance of pediatric work is scarce [17, 18].

All reimbursement experts, of all countries, have difficulty with assessing the added benefit for pediatric indications due to insufficient data. Heterogeneity in approval requirements for regulatory and reimbursement decisions are common, and as HTA bodies are often not involved earlier in product development processes these are often not considered and addressed early enough. This discrepancy and heterogeneity generate significant challenges in developing further evidence after marketing authorization [19]. While FR is the only country with specific pediatric incentives for reimbursement, the lack of awareness among developers could potentially create challenges. Literature validates these findings, particularly demonstrating that French pricing is often higher compared to other European countries [20].

All countries except FR treat pediatric indications identically to adult indications. However, NL seems less attractive for launching PM due to the small size of the country and complexity of the process. Literature confirms that most of the challenges of Dutch pricing and reimbursement include patient access, pricing models, and affordability aspects [21].

Interviews suggested that UK grants immediate reimbursement for PM if there is reimbursement already agreed for the adult indication, which is also confirmed by the NHS regulation [22]. DE grants immediate reimbursement after EU approval irrespective of the adult indication. However, due to the relatively small population, the price resulting from the negotiation after the free pricing period tends to be lower because the comparator is often a generic medicine with a long data history [23]. Industry criticizes that reimbursement authorities are demanding data that cannot be available at the early stages of market entry of an innovative product [23].

While most of the countries face the same methodological challenges regarding cost-effectiveness and quality-of-life measurements, UK and FR include patient representatives, who potentially provide lived experience data unavailable in the published literature. According to literature, it can be concluded that the nation’s reimbursement system seems to experience the same challenges as NL. Namely, difficulty of assessing comparative effectiveness related to comparators, absence of specific pediatric quality-of-life questionnaires and biases involving children into these, and variability of reimbursement for different pediatric age groups. The latter challenge was confirmed by experts in HTA bodies in Europe and the UK. These experts explained that patients may receive reimbursement for a treatment in their early life years but later once they turn eighteen due to more effective treatments available in that age group and the original indication not being applicable for them anymore. Several research groups are currently working on the measurement quality of life in children, including development of questionnaires [24]. Literature explains that due to the constant cost-per-QALY thresholds over the years, the ability to receive a positive recommendation when introducing certain new products in an expanding market is difficult [25]. In addition, guidance from major health authorities is often discordant, requiring country/region-specific development that may bring study/program feasibility into question due to additional costs and therefore the paucity of approved/labeled pediatric medicines.

This study is particularly relevant due to the recent advancements in regenerative medicine (e.g., cell therapies or gene editing constructs) that are predominantly targeted for pediatric populations and largely confirms previous experts’ recommendations [26, 27]. First, more emphasize on multi-stakeholder collaboration must be put where objectives from all stakeholders are communicated. Second, collaborating to develop joint databases and post-approval monitoring procedures using real-world evidence could be used to confirm the added benefit for pediatric indications in the market over time. Third, early dialogue including HTA bodies when discussing the PIP could streamline the heterogeneity in requirements between regulators and HTA bodies and identify the unmet medical needs in pediatrics based on a mechanism of action of a new molecule [26]. One initiative that has been recently developed is the Oslo Medicines Initiative (OMI) which sought to bring key players together, create a neutral platform for dialogue and collaboration among these players, build trust between stakeholders, and identify pragmatic solutions to increase access to medicines in the European Region [28]. If applied to pediatric market access, this initiative could enhance multi-stakeholder collaboration and early dialogue. Another initiative that is in line with the proposed solutions is the Joint Clinical Assessment of Efficacy by EUnetHTA. As of 2023, this initiative will be used to assess all oncology drugs and advanced therapy medicinal products and could potentially offer a solution to pediatric medicines as well [29].

### Limitations and Themes for Future Research

This study holds several limitations. First, only one researcher performed data analysis and coding due to time restrictions. This leads to a limited analysis with limited understanding of different patterns and the ability in attributing them to the factors identified. More comprehensive results could have been achieved by performing the data analysis with two researchers. Second, although reimbursement experts were interviewed for all countries in industry, this study does not include HTA representatives of FR and DE. Therefore, confirmation by German and French regulatory representatives of challenges is lacking. Third, as two experts preferred to conduct the interview in Dutch, some translational biases could have occurred during the data analysis. Fourth, even though data saturation was reached in the majority of expert groups and key experts were selected, the research was executed with a restricted cohort of participants who provided responses in their individual capacity, which could lead to presence of possible selection biases and lack of generalizability. Lastly, besides that some selection bias could have occurred, this study exclusively concentrates on British, Dutch, French, and German experts, it should be recognized that different countries may have different requirements.

Themes for future research include (a) the effectiveness of the joint clinical assessment with regards to the economic capabilities of individual countries; (b) the effect of extrapolation and real-world evidence on uncertainty in PM development after market approval; (c) the relative access to appropriate PM in each country’s jurisdiction and assess differences, and (d) the difference in prescribing, reimbursing, and using pediatric indications that have an adult indication compared to those that have not been approved and thus used off-label.

## Conclusion

The results of this study encourage developing specific pricing and reimbursement models based on the economic capabilities of the country and tailored to pediatric data. The next step for all stakeholders should be to fully engage in multi-stakeholder discussions and collaboration to anticipate evidence needs and ensure facilitated market access. Early collaboration between all stakeholders, more transparency of requirements, and involvement of patient representatives in decision-making could reduce delays and challenges and strengthen pediatric market access. Additional pediatric incentives, such as, for example, in FR, should be taken as best practice. Themes for further testing connected to the expressed challenges include clinical trials that are designed for smaller sample sizes and heterogeneity such as orphan drugs, more hands-on guidance for parents to make better quality informed consent decisions, pediatric pricing irrespective of adult indications, managed access to pediatric adaptations of adult medicines, and inclusion of real-world monitoring and data collection for proposed pediatric formulations.
